# Prediction of treatment failure and compliance in patients with tuberculosis

**DOI:** 10.1186/s12879-020-05350-7

**Published:** 2020-08-24

**Authors:** Hyeon-Kyoung Koo, Jinsoo Min, Hyung Woo Kim, Joosun Lee, Ju Sang Kim, Jae Seuk Park, Sung-Soon Lee

**Affiliations:** 1 Division of Pulmonary and Critical Care Medicine, Department of Internal Medicine, Ilsan Paik Hospital, Inje University College of Medicine, Juhwa-ro 170, Ilsanseo-gu, Goyang, 10380 Republic of Korea; 2 Division of Pulmonary and Critical Care Medicine, Department of Internal Medicine, Daejeon St. Mary’s Hospital, College of Medicine, The Catholic University of Korea, Seoul, Republic of Korea; 3 Division of Pulmonary and Critical Care Medicine, Department of Internal Medicine, Incheon St. Mary’s Hospital, College of Medicine, The Catholic University of Korea, Seoul, Republic of Korea; 4 Division of TB Epidemic Investigation, Korea Centers for Disease Control and Prevention, Osong, Republic of Korea; 5 Division of Pulmonary Medicine, Department of Internal Medicine, Dankook University College of Medicine, Cheonan, Republic of Korea

**Keywords:** Public-private sector partnership, Treatment failure, Tuberculosis, Korea

## Abstract

**Background:**

To improve treatment outcomes for tuberculosis (TB), efforts to reduce treatment failure are necessary. The aim of our study was to describe the characteristics of subjects who had failed treatment of tuberculosis and identify the risk factors for treatment failure and poor compliance using national data.

**Methods:**

A multicenter cross-sectional study was performed on tuberculosis subjects whose final outcome was reported as treatment failure during 2015–2017. The same number of subjects with treatment success during the same study period were randomly selected for comparison. Demographics, microbiological, radiographic, and clinical data were collected based on in-depth interviews by TB nurse specialists at all Public Private Mix (PPM) participating hospitals in South Korea.

**Results:**

A total of 52 tuberculosis patients with treatment failure were enrolled. In a multivariable analysis, the presence of diabetes, previous history of tuberculosis, and cavity were identified as risk factors for treatment failure; and Medicaid support was a favorable factor for treatment success (area under the curve [AUC]: 0.79). Age, low body mass index (BMI), presence of diabetes, preexisting lung disease, positive sputum acid-fast bacilli (AFB) smear result, and the presence of multidrug-resistant tuberculosis (MDR-TB) were significantly associated with presence of cavities. Younger age, lower BMI and previous history of TB were associated with poor compliance during treatment (AUC: 0.76).

**Conclusion:**

To reduce treatment failure, careful evaluation of the presence of diabetes, previous TB history, underlying lung disease, cavity, results of sputum AFB smears, and socioeconomic status are needed. To enhance treatment compliance, more attention should be paid to younger patients with lower BMIs during follow-up.

## Background

Tuberculosis (TB) remains an unsolved public health problem despite the strenuous efforts exerted by numerous countries [1]. In spite of the high economic status and medical services system, South Korea is included as an intermediate TB burden country and is in first place for TB development among the countries that are members of the Organization for Economic Co-operation and Development [2]. To overcome this situation, the Public Private Mix (PPM) collaboration model that supports diagnosis and treatment of TB has been implemented as a national TB control strategy, and as a consequence, the rate of TB has been decreasing. However, a considerable rate of treatment failure still exists, complicated by the development of drug resistance, and disease-related morbidities and mortalities that prevent the curing of TB. To triumph in battling tuberculosis, further efforts to decrease poor outcomes such as treatment failure and increase treatment compliance are needed. The clinical characteristics of those who had failed treatment are limited because of difficulties in collecting appropriate subjects. In previous studies, human immunodeficiency virus (HIV) co-infection, previous history of TB, sputum smear positivity after 2 months of treatment, male sex, young or advanced age, drug resistance, and residence in a solitary area have been proposed as risk factors for poor outcome [3–9]. However, such studies were performed on a small number of patients in high-TB burden countries with limited medical resources. Because South Korea has a different socioeconomic environment, a low rate of HIV infection, and high access to medical services [10, 11], a different strategy to control TB is required in South Korea.

The aim of our study was to describe the subjects´ characteristics and to identify the risk factors of treatment failure and poor compliance for the purpose of predicting risk groups to improve treatment outcomes. Collecting enough treatment failure cases from individual institutions was difficult; therefore, we collected national data of treatment failure cases from all the PPM-participating hospitals.

## Methods

In South Korea, physicians must notify the diagnosis and treatment of TB when they initially diagnose or suspect TB and multidrug-resistant tuberculosis (MDR-TB). Under the PPM project, all patients are followed during treatment until the report of final treatment outcomes by TB nurse specialists dispatched to private PPM hospitals. TB nurse specialists record information about medications, side effects, and compliance with the Korean National TB Surveillance System of PPM hospitals [12]. More than 210 TB nurse specialists at 127 PPM hospitals and 236 public health officials at 254 public health centers across the country work under the PPM projects. Approximately 69% of new TB patients were treated at PPM hospitals in 2017. From January 2015 to December 2017, data of subjects who failed TB treatment were collected from all the PPM participating hospitals and TB nurse specialists at each hospital completed treatment failure case report forms. Treatment failure was defined as remaining culture-positive after 4 months of treatment or at the end of treatment [13]. Patients who took at least 95% of medication as prescribed by the clinician were defined to be compliance group [4]. Baseline characteristics such as age, sex, body mass index (BMI), respiratory symptoms, previous history of TB, co-existing comorbidities, and smoking and alcohol history were recorded. Furthermore, results of radiographic, microbiological, and clinical data, including sputum smear, drug susceptibility test, treatment regimen, and treatment compliance, were retrospectively collected by TB nurse specialists. Presence or absence of cavity were judged by the results of chest X-ray or chest computed tomography. After collecting information about treatment failure cases, the same number of subjects with treatment success during the same study period were randomly selected and the characteristics of these two groups were compared. Additionally, the characteristics of treatment compliance and noncompliance groups were also compared. Based on the results, a prediction model for treatment failure was constructed.

### Statistical analysis

The subjects´ characteristics were presented as the mean and standard deviation for continuous variables and as relative frequencies for categorical variables. Statistical analysis was performed using R (version 3.6.0). Means were compared using a t-test or analysis of variance (ANOVA), and categorical variables were compared using a chi-squared test or Fisher’s exact test. To construct early prediction model, logistic regression was performed including significant variables in univariable analysis at *P* value < 0.1 in addition to age, sex, and BMI; variables that could be measured at the beginning of TB treatment such as respiratory symptoms, previous history of TB, social history, comorbidities, radiographic characteristic, and sputum acid-fast bacilli (AFB) smear results were included. The best model was selected with the stepwise selection method using stepAIC function in MASS package. To compare the classification ability of each model, the area under the curve (AUC) of the receiver operating characteristic curve (ROC) was calculated using the ROCR package. To assess predictive validity, leave-one-out cross validation (LOOCV) using boot package was performed.

### Ethical approval

The study was conducted in accordance with the Declaration of Helsinki. The Korea Centers for Disease Control and Prevention (KCDC) has the authority to hold and analyze surveillance data for public health and research purposes. KCDC approved the data use and provided data without personal identification information.

## Results

### Baseline characteristics

A total of 52 subjects who failed treatment and 50 who had treatment success during the study period were enrolled. The demographic and clinical characteristics of these participants are summarized in Table 1. The mean age was 44.5 years, and 38 (73.1%) were men. Among them, 29 (55.8%) were newly diagnosed cases, 12 (23.1%) recurred cases, 7 (13.5%) re-treated cases after failure, and 3 (5.8%) re-treated cases after treatment cessation. Twenty-seven (51.9%) were AFB smear-positive. The initial treatment regimen was as follows: HREZ was administered to 40 (76.9%) patients, HRE was administered to 1 (1.9%) patient, and 11 (21.2%) patients were administered others. The demographic and clinical characteristics of the randomly selected subjects who succeeded during the same study period, are also summarized in Table 1. Age, sex, BMI, and smoking and alcohol history were not significantly different between the two groups. However, the presence of diabetes mellitus, previous history of TB, and sputum AFB smear positivity were increased in the treatment failure group. In addition, the cavity was found more frequently on chest radiography in the treatment failure group. Compared to subjects without cavities, those with cavity presented with more underlying lung disease, diabetes, and positive sputum AFB smear results. Detailed characteristics comparing these groups are summarized in Supplemental Table S1. Nineteen (36.5%) subjects in the treatment failure group had MDR-TB. Fifty (28.8%) subjects presented non-compliance during treatment. A comparison of the characteristics between the treatment compliant and noncompliant groups is summarized in Supplemental Table S2. Noncompliant subjects were younger (*P* = 0.02) and less obese (*P* = 0.02) than compliant subjects. A previous history of TB was less frequent in the noncompliant group. There was no association between age and BMI in both sex (*P* = 0.12, Supplemental Figure S1). The relative distribution of overlaps among subjects who failed treatment, presented with MDR-TB, and were noncompliant are summarized in Supplemental Figure S2.

**Table 1:** Clinical characteristics comparing treatment success group and treatment failure group

	Treatment success (***N*** = 50)	Treatment failure (***N*** = 52)	***P***-value
**Age**	44.0(31.5–65)	44.5(29.5–67)	0.84
**Male sex**	28(56.0%)	38(73.1%)	0.11
**BMI**	21.1 ± 2.88	20.9 ± 3.07	0.72
**Smoking**			0.67
Current	10(20.0%)	13(25.0%)	
Ex-	10(20.0%)	7(13.5%)	
Never	30(60.0%)	32(61.5%)	
**Drinking**			0.21
Heavy	3(6.0%)	3(5.8%)	
Social	19(38.0%)	19(36.5%)	
None	24(48.0%)	30(57.7%)	
**Underlying disease**			
Diabetes	5(10.0%)	4(26.9%)	0.041
Lung ds	3(6.0%)	4(7.7%)	> 0.99
Heart ds	1(2.0%)	3(5.8%)	0.24
Liver ds	2(4.0%)	1(1.9%)	> 0.99
Kidney ds	1(2.0%)	3(5.8%)	0.36
Prev TB Hx	8(16.0%)	19(36.5%)	0.01
**Symptom**			
Cough/sputum	20(40.0%)	29(55.8%)	0.16
Dyspnea	10(20.0%)	7(13.5%)	0.43
Chest pain	7(14.0%)	3(5.8%)	0.20
Hemoptysis	1(2.0%)	3(5.8%)	0.62
Fever	5(10.0%)	3(5.8%)	0.48
Weakness	0(0%)	1(1.9%)	> 0.99
Weight loss	6(12.0%)	4(7.7%)	0.52
Asymptomatic	17(34.0%)	15(28.8%)	0.73
**Enrollment**			
Newly diagnosed	42(84.0%)	29(55.8%)	
Recurred	8(16.0%)	12(23.1%)	
Retreatment	0(0%)	7(13.5%)	
Default	0(0%)	3(5.8%)	
**Social Hx**			
Occupation	18(36.0%)	23(44.2%)	0.62
Marriage	28(56.0%)	33(63.5%)	0.13
Medicaid	6(12.0%)	1(1.9%)	0.06
**CPA**			
Normal	4(8.0%)	2(3.8%)	0.43
Cavity (+)	8(17.0%)	26(50.0%)	0.001
Unilateral	26(52%)	23(44.2%)	0.30
Bilateral	17(34%)	27(51.9%)	
**AFB smear**			
Positive	11(23.4%)	27(51.9%)	
**DST**			< 0.001
All sensitive	46(92.0%)	21(40.4%)	
R to INH	2(4.0%)	5(9.6%)	
R to RFP	1(2.0%)	5(9.6%)	
Any other R	1(2.0%)	2(3.8%)	
**Treatment regimen**			
HREZ	38(76.0%)	40(76.9%)	
HRE	11(22.0%)	1(1.9%)	
Others	1(2.0%)	11(21.2%)	
**Drug compliance**			
Good compliance	50(100%)	37(71.2%)	
Poor compliance	0(0%)	15(28.8%)	

*BMI* Body mass index, *ds* Disease, *Hx* History, *R* Resistance, *INH* Isoniazid, *RFP* Rifampin, *MDR* Multi-drug resistance

### Prediction of treatment failure and noncompliance

**Fig. 1:**
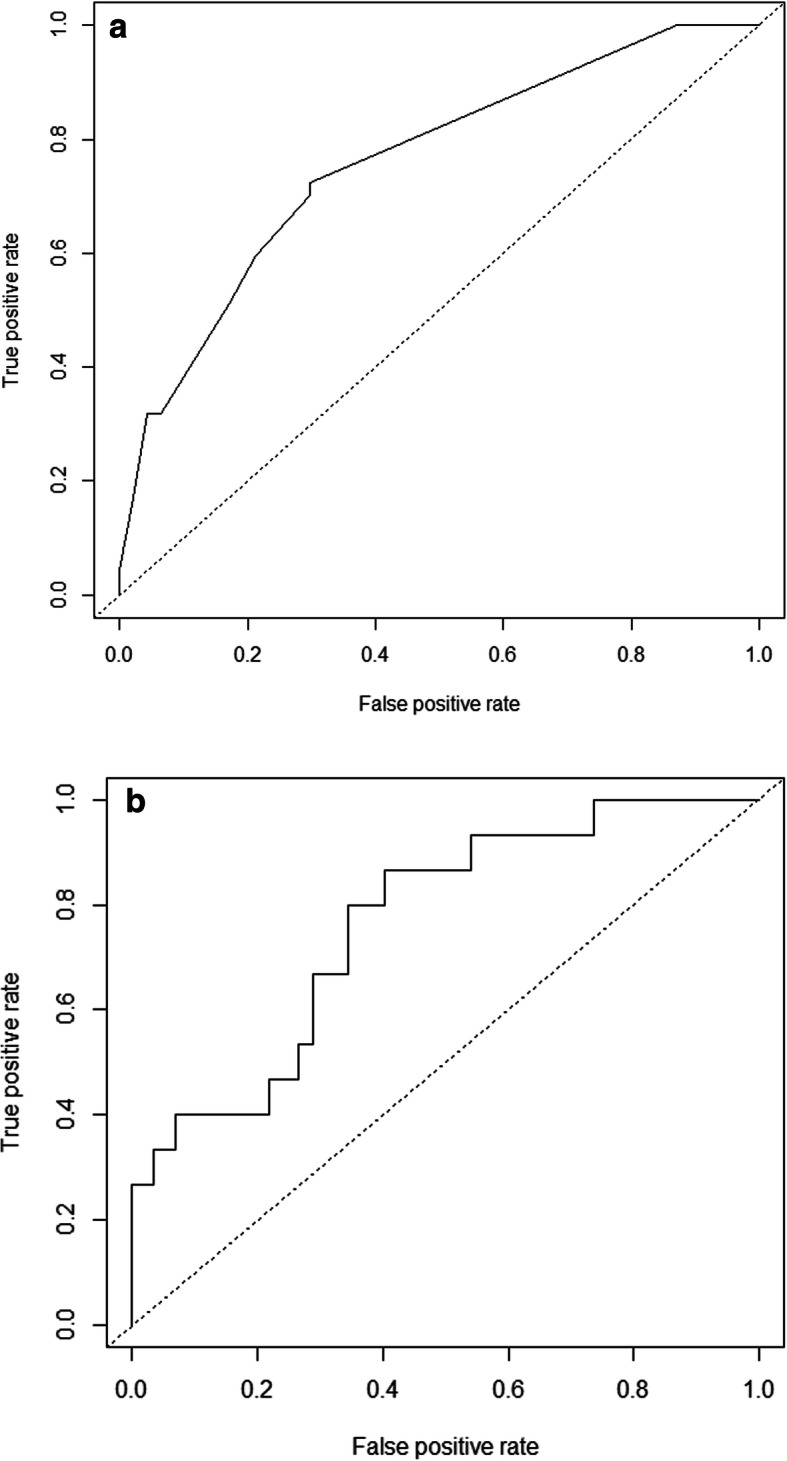
Receiver operating curve for predicting treatment failure (**a**) and non-compliance to treatment (**b**). Figure legends: (**a**) AUC: 0.79, (**b**) AUC: 0.76

For multivariable analysis, variables of age, sex, presence of diabetes, previous history of TB, Medicaid support, presence of cavity, and positivity of sputum AFB smear were included; the presence of diabetes mellitus, previous history of TB, presence of cavity, and absence of Medicaid support were selected as independent risk factors for treatment failure in all study subjects (Table 2). The AUC of the ROC of this model was 0.792 (Fig. 1a). The predictive validity using LOOCV of this model was 0.795. Younger age, lower BMI, presence of diabetes, presence of preexisting lung disease, positivity of sputum AFB smear, and presence of MDR-TB were significantly associated with the presence of cavities (Table 2). In subjects without MDR-TB, the presence of diabetes (odds ratio [OR] = 3.58, 95% confidence interval [CI]: 0.97–13.23), previous history of TB (OR = 3.45, 95% CI: 1.39–8.54), and presence of cavity (OR = 2.74, 95% CI: 0.90–8.36) were selected for predicting treatment failure, and the AUC of the ROC curve for this model was 0.73 (Supplemental Figure S3). For compliance during treatment, variables of age, sex, BMI, and previous history of TB were included for multivariable analysis. Younger age, lower BMI, and previous history of TB were associated with poor drug compliance, and the AUC of the ROC curve for this model was 0.765 (Fig. 1b). The predictive validity of this model was 0.887.

**Table 2:** Multivariable analysis for treatment failure, presence of cavity, and treatment compliance

	Risk factors	Odds ratio	95% CI
**Treatment failure**	Diabetes mellitus	3.25	0.89–11.91
	Previous history of TB	2.32	0.72–7.41
	Medicaid	0.11	0.01–1.29
	**Presence of Cavity**	4.78	1.72–13.23
**Cavity**	Age	0.957	0.92–0.99
	Body mass index	0.824	0.67–1.01
	Diabetes mellitus	3.897	0.97–15.59
	Pre-existing lung disease	13.373	1.37–130.22
	Sputum smear positive	6.730	2.17–20.90
	MDR-TB	6.927	1.66–28.84
**Poor compliance**	Age	0.964	0.93–0.99
	Body mass index	0.773	0.59–1.00
	Previous history of TB	2.116	1.11–4.05

*TB* Tuberculosis, *MDR* Multi-drug resistance

## Discussion

In our study, we compared the characteristics of patients with treatment failure with those of treatment success and built a prediction model for treatment failure with high predictive power. The presence of diabetes, previous history of TB, and cavity were independent risk factors for treatment failure, and Medicaid support was favorable one for treatment success. For the presence of cavities, younger age, low BMI, diabetes, preexisting lung disease, positive sputum AFB smear, and MDR-TB were independent risk factors. Since treatment compliance is an essential component of treatment success, younger age, lower BMI, and previous history of TB were unfavorable predictors for compliance, and these predictors were connected to each other acting as complicated effect modifiers.

The first model for predicting treatment failure by Kalhori et al. [14] used clinical data including old age, male sex, body weight, nationality, prisoner status, and previous history of TB, and achieved an AUC of 0.70. Recently, Sauer et al. [15] tried to predict treatment failure by machine learning using demographic and laboratory data and reported a best AUC of 0.74. However, this model lacked information about comorbidities; our model included such variables and yielded considerably high prediction power, an AUC of 0.79. Furthermore, our model was constructed based on routinely collected data we recruited retrospectively that were easily gathered in clinical practice.

The remarkable traits to review carefully are the presence of diabetes and age. Diabetes is known to be associated with the development of TB, possibly mediated by several mechanisms of proinflammatory cytokine [16–20], especially if diabetes-related complications co-exist [21]. In our study, we identified that diabetes was not only related to the development of TB, but also related to treatment failure and the presence of cavities. Older age is a well-known risk factor for the development of TB and higher TB-related death rates [22, 23]. However, younger age was related to the presence of cavity, and poor compliance to treatment. Furthermore, there have been reports that BMI is inversely associated with the risk of TB [24]. Obesity presented a protective effect, while a lower BMI was associated with the development of TB [25] and higher TB-related mortality [26]. However, BMI is also associated with metabolic syndrome such as diabetes mellitus [27], so these opposite effects of BMI could confuse their role in TB [25]. In our study population, BMI in subjects with diabetes was not significantly different that in those without diabetes (20.75 vs. 22.30; *P* = 0.13); instead, lower BMI was related to the presence of cavity and poor compliance to treatment. Medicaid support was associated with more treatment success and ensured the importance of national efforts such as the PPM program in defeating tuberculosis.

Although our study revealed the complex associations of several risk factors, there are limitations that should be noted when interpreting our results. This study is a retrospective case-control study, and data were recruited after results of the sputum culture reports came out, so there could be some missing information for each variable. The problem of recall bias from patients, families, and TB nurse specialists may exist. Though we tried to collect cases, especially focusing on non MDR-TB patients, as a complete enumeration among PPM participating hospital, the number of enrolled patients was small; which reflects the frequency of the treatment failure in South Korea. Post hoc power analysis estimates power of our study as 0.767 if we assume medium to large effect size of 0.45. However, further large prospective cohort studies to confirm our findings are necessary. Additionally, these cases were recruited from PPM participating hospitals, and although approximately 70% of TB patients are treated under the PPM program, this could limit the generalizations of our study.

## Conclusions

In conclusion, in order to reduce treatment failure, the presence of diabetes, previous history of TB, underlying lung disease, sputum AFB smear results, and socioeconomic status should be carefully evaluated, and more attention has to be given to younger patients with lower BMIs during follow-up to improve treatment compliance. Further, larger studies are needed in order to confirm our findings.

## Supplementary information

**Additional file 1: Table S1.** Comparison of characteristics between subjects with and without cavitary lesion on chest radiography. **Table S2.** Comparison of characteristics between compliant and non-compliant subjects. **Figure S1.** No association between age and body mass index in both sexes. **Figure S2.** Overlapping of non-compliance and presence of multi-drug resistance tuberculosis among subjects of treatment failure. **Figure S3.** Receiver operating curve for predicting treatment failure in subjects without multidrug resistant tuberculosis.

## Data Availability

The ownership of the primary dataset lies with the Korea Centers for Disease Control and Prevention (KCDC). The datasets used and/or analyzed during the present study are available on reasonable request after obtaining permission from the KCDC in advance.

## Abbreviations

AFB: Acid-fast bacilli
BMI: Body mass index
CI: Confidence interval
MDR-TB: Multidrug-resistant tuberculosis
OR: Odds ratio
PPM: Public Private Mix
ROC: Receiver operating characteristic curve
TB: Tuberculosis
AUC: Under the curve
