# Supplementary material for: Prediction of treatment failure and compliance in patients with tuberculosis
Source: BMC Infect Dis. 2020 Aug 24;20:622. doi: 10.1186/s12879-020-05350-7 (PMC7446045; doi:10.1186/s12879-020-05350-7)

**Supplements**

Supplemental Table S1. Comparison of characteristics between subjects with and without cavitary lesion on chest radiography

|  |  | **Cavity (+)**  **(N= 34)** | **Cavity (-)**  **(N=63)** | **P-value** |
| --- | --- | --- | --- | --- |
| **Age** |  | 42.0(30.25-61.75) | 50.0(30.5-70.5) | 0.49 |
| **Male sex** |  | 24(70.6%) | 37(59.7%) | 0.38 |
| **BMI** |  | 20.39 ± 3.56 | 21.09 ± 2.56 | 0.27 |
| **Smoking** |  |  |  | 0.88 |
|  | Current | 7(20.6%) | 6(17.6%) |  |
|  | Ex- | 6(17.6%) | 9(14.5%) |  |
|  | Never | 21(61.8%) | 38(61.3%) |  |
| **Drinking** |  |  |  | 0.29 |
|  | Heavy | 1(2.9%) | 3(4.8%) |  |
|  | Social | 11(32.4%) | 26(41.9%) |  |
|  | None | 22(64.7%) | 30(48.4%) |  |
| **Underlying disease** | |  |  |  |
|  | Diabetes | 10(29.4%) | 8(12.5%) | 0.08 |
|  | Lung ds | 5(14.7%) | 2(3.1%) | 0.04 |
|  | Heart ds | 2(5.9%) | 2(3.1%) | 0.73 |
|  | Liver ds | 0(0%) | 3(4.7%) | 0.55 |
|  | Kidney ds | 2(5.9%) | 2(3.1%) | 0.59 |
|  | Prev TB Hx | 11(32.4%) | 12(18.8%) | 0.12 |
| **Symptom** | |  |  |  |
|  | Cough/sputum | 18(52.9%) | 29(46.0%) | 0.77 |
|  | Dyspnea | 5(14.7%) | 11(17.5%) | 0.80 |
|  | Chest pain | 4(11.8%) | 6(9.5%) | 0.73 |
|  | Hemoptysis | 2(5.9%) | 2(3.2%) | 0.60 |
|  | Fever | 2(5.9%) | 6(9.5%) | 0.72 |
|  | Weakness | 0(0%) | 1(1.6%) | >0.99 |
|  | Weight loss | 1(2.9%) | 9(14.3%) | 0.16 |
|  | Asymptomatic | 12(35.3%) | 18(28.6%) | 0.65 |
| **Social Hx** | |  |  |  |
|  | Occupation | 16(47.1%) | 37(58.7%) | 0.66 |
|  | Marriage | 12(35.3%) | 21(33.3%) | 0.18 |
|  | Family | 3(8.8%) | 20(31.7%) | 0.01 |
|  | Medicaid | 2(5.9%) | 5(7.9%) | >0.99 |
| **CPA** |  |  |  | 0.24 |
|  | Unilateral | 9(26.5%) | 45(71.4%) |  |
|  | Bilateral | 12(35.3%) | 17(27.0%) |  |
| **AFB smear** | |  |  | <0.001 |
|  | Positive | 23(67.6%) | 14(21.9%) |  |
| **MDR** |  |  |  | <0.001 |
|  |  | 14 (41.2%) | 4 (6.3%) |  |
| **Mode of development** | |  |  | 0.03 |
|  | New case | 19(55.9%) | 51(81.0%) |  |
|  | Recurred | 8(23.5%) | 9(14.3%) |  |
|  | Retreatment | 5(14.7%) | 1(1.6%) |  |
|  | Default | 1(2.9%) | 1(1.6%) |  |
| **Treatment regimen** | |  |  | 0.001 |
|  | HREZ | 24(70.6%) | 44(73.3%) |  |
|  | HRE | 1(2.9%) | 11(18.3%) |  |
|  | Others | 9(26.5%) | 8(12.7%) |  |
| **Treatment compliance** | |  |  | 0.56 |
|  | Good | 28(82.4%) | 54(85.7%) |  |
|  | Poor | 6(17.6%) | 9(14.3%) |  |

BMI, body mass index; ds, disease; Hx, history; R, resistance; INH, isoniazid; RFP, rifampin; MDR, multi-drug resistance

Supplemental Table S2. Comparison of characteristics between compliant and non-compliant subjects

|  |  | **Tx success**  **(N=50)** | **Good compliance**  **(N=37)** | **Poor compliance**  **(N=15)** | **P-value** |
| --- | --- | --- | --- | --- | --- |
| **Age** |  | 44.0(31-66) | 52.0±20.2 | 37.5±18.1 | 0.02 |
| **Male sex** |  | 28(56.0%) | 27(73.0%) | 11(73.3%) | >0.99 |
| **BMI** |  | 21.1 ± 2.88 | 21.5 ± 3.1 | 19.3 ± 2.5 | 0.02 |
| **Smoking** |  |  |  |  | 0.93 |
|  | Current | 10(20.0%) | 9(24.3%) | 4(26.7%) |  |
|  | Ex- | 10(20.0%) | 5(13.5%) | 2(13.3%) |  |
|  | Never | 30(60.0%) | 23(62.2%) | 9(60.0%) |  |
| **Drinking** |  |  |  |  | >0.99 |
|  | Heavy | 3(6.0%) | 2(5.4%) | 1(6.7%) |  |
|  | Social | 19(38.0%) | 13(35.1%) | 6(40.0%) |  |
|  | None | 24(48.0%) | 22(59.5%) | 8(53.3%) |  |
| **Underlying disease** | |  |  |  |  |
|  | Diabetes | 5(10.0%) | 12(32.4%) | 2(14.3%) | 0.73 |
|  | Lung ds | 3(6.0%) | 4(10.8%) | 0(0%) | 0.59 |
|  | Heart ds | 1(2.0%) | 3(8.1%) | 0(0%) | 0.70 |
|  | Liver ds | 2(4.0%) | 1(2.7%) | 0(0%) | >0.99 |
|  | Kidney ds | 1(2.0%) | 3(8.3%) | 0(0%) | >0.99 |
|  | Prev TB Hx | 8(16.0%) | 16(43.2%) | 3(21.4%) | <0.001 |
| **Symptom** | |  |  |  |  |
|  | Cough/sputum | 20(40.0%) | 19(51.4%) | 10(66.7%) | 0.37 |
|  | Dyspnea | 10(20.0%) | 4(10.8%) | 3(20.0%) | >0.99 |
|  | Chest pain | 7(14.0%) | 0(0%) | 3(20.0%) | 0.33 |
|  | Hemoptysis | 1(2.0%) | 2(5.4%) | 1(6.7%) | >0.99 |
|  | Fever | 5(10.0%) | 3(8.1%) | 0(0%) | 0.48 |
|  | Weakness | 0(0%) | 1(2.7%) | 0(0%) | >0.99 |
|  | Weight loss | 6(12.0%) | 3(8.1%) | 1(6.7%) | >0.99 |
|  | Asymptomatic | 17(34.0%) | 13(35.1%) | 2(13.3%) | 0.18 |
| **Social Hx** | |  |  |  |  |
|  | Occupation | 18(36.0%) | 15(40.5%) | 8(53.3%) | 0.70 |
|  | Marriage | 28(56.0%) | 25(67.6%) | 8(53.3%) | 0.44 |
|  | Family | 31(62.0%) | 32(86.5%) | 13(86.7%) | 0.79 |
|  | Medicaid | 6(14.0%) | 1(2.7%) | 0(0%) | 0.56 |
| **CPA** |  |  |  |  |  |
|  | Cavity (+) | 8(17.0%) | 20(54.1%) | 6(40.0%) | 0.25 |
|  | Unilateral | 26(56.5%) | 16(44.4%) | 7(46.7%) |  |
|  | Bilateral | 17(37.0%) | 20(55.6%) | 7(46.7%) | 0.28 |
| **AFB smear** | |  |  |  | 0.37 |
|  | Positive | 11(23.4%) | 21(56.8%) | 6(40.0%) |  |
| **MDR** |  |  |  |  | >0.99 |
|  |  | 0(0%) | 12(36.4%) | 5(38.5%) |  |
| **DST** |  | 37(74.0%) | 31 | 15 | 0.42 |
|  | All S |  | 11(35.5%) | 4(26.7%) |  |
|  | Any R |  | 2(6.5%) | 2(13.3%) |  |
|  | R to H | 2(4.0%) |  |  |  |
|  | R to R | 1(2.0%) | 1(3.2%) | 1(6.7%) |  |
|  | MDR |  | 13(35.1%) | 6(40%) |  |
| **Mode of development** | |  |  |  | 0.11 |
|  | New case | 42(84.0%) | 20(54.1%) | 9(60.0%) |  |
|  | Recurred | 8(16.0%) | 9(24.3%) | 3(20.0%) |  |
|  | Retreatment | 0(0%) | 7(18.9%) | 0(0%) |  |
|  | Default | 0(0%) | 1(2.7%) | 2(13.3%) |  |
| **Treatment regimen** | |  |  |  | 0.01 |
|  | HREZ | 38(76.0%) | 30(81.1%) | 5(38.5%) |  |
|  | HRE | 11(22.0%) | 2(5.4%) | 0(0%) |  |
|  | Others | 1(2.0%) | 3(9.1%) | 5(45.5%) |  |

BMI, body mass index; ds, disease; Hx, history; R, resistance; INH, isoniazid; RFP, rifampin; MDR, multi-drug resistance

Supplemental Figure S1. No association between age and body mass index in both sexes


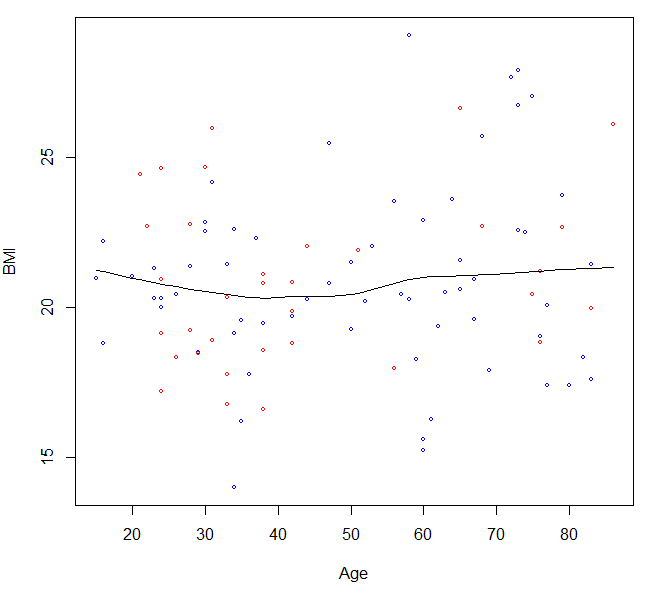


Blue dots mean male sex and red dots mean female sex

Supplemental Figure S2. Overlapping of non-compliance and presence of multi-drug resistance tuberculosis among subjects of treatment failure


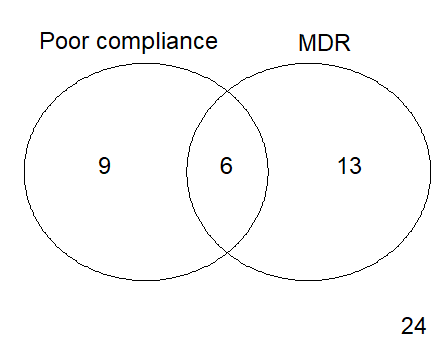


MDR, multidrug-resistance

Supplemental Figure S3. Receiver operating curve for predicting treatment failure in subjects without multidrug resistant tuberculosis


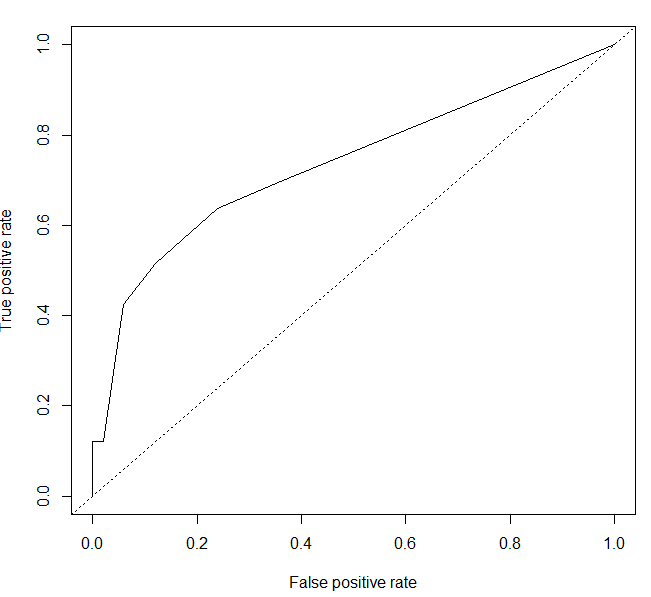

Supplement: Supplementary file 1 — Additional file 1: Table S1. Comparison of characteristics between subjects with and without cavitary lesion on chest radiography. Table S2. Comparison of characteristics between compliant and non-compliant subjects. Figure S1. No association between age and body mass index in both sexes. Figure S2. Overlapping of non-compliance and presence of multi-drug resistance tuberculosis among subjects of treatment failure. Figure S3. Receiver operating curve for predicting treatment failure in subjects without multidrug resistant tuberculosis. [file 12879_2020_5350_MOESM1_ESM.docx]
